# Developing an early screening instrument for predicting psychological morbidity after critical illness

**DOI:** 10.1186/cc13018

**Published:** 2013-09-24

**Authors:** Anna Schandl, Matteo Bottai, Elisabeth Hellgren, Orjan Sundin, Peter V Sackey

**Affiliations:** 1 Department of Anesthesiology, Surgical Services and Intensive Care Medicine, Karolinska University Hospital Solna, 171 76 Stockholm, Sweden; 2 Section for Anesthesiology and Intensive Care Medicine, Department of Physiology and Pharmacology, Karolinska Institutet, 171 77 Stockholm, Sweden; 3 Unit of Biostatistics, Department of Environmental Medicine, Karolinska Institutet, 171 77 Stockholm, Sweden; 4 Department of Psychology, Division of Social Sciences, Mid Sweden University, 831 25 Östersund, Sweden

## Abstract

**Introduction:**

Guidelines recommend follow-up for patients after an intensive care unit (ICU) stay. Methods for identifying patients with psychological problems after intensive care would be of value, to optimize treatment and to improve adequate resource allocation in ICU follow-up of ICU survivors. The aim of the study was to develop a predictive screening instrument, for use at ICU discharge, to identify patients at risk for post-traumatic stress, anxiety or depression.

**Methods:**

Twenty-one potential risk factors for psychological problems - patient characteristics and ICU-related variables - were prospectively collected at ICU discharge. Two months after ICU discharge 252 ICU survivors received the questionnaires Post-Traumatic Stress Symptom scale -10 (PTSS-10) and Hospital Anxiety and Depression Scale (HADS) to estimate the degree of post-traumatic stress, anxiety and depression.

**Results:**

Of the 150 responders, 46 patients (31%) had adverse psychological outcome, defined as PTSS-10 >35 and/or HADS subscales ≥8. After analysis, six predictors were included in the screening instrument: major pre-existing disease, being a parent to children younger than 18 years of age, previous psychological problems, in-ICU agitation, being unemployed or on sick-leave at ICU admission and appearing depressed in the ICU. The total risk score was related to the probability for adverse psychological outcome in the individual patient. The predictive accuracy of the screening instrument, as assessed with area under the receiver operating characteristic curve, was 0.77. When categorizing patients in three risk probability groups - low (0 to 29%), moderate (30 to 59%) high risk (60 to 100%), the actual prevalence of adverse psychological outcome in respective groups was 12%, 50% and 63%.

**Conclusion:**

The screening instrument developed in this study may aid ICU clinicians in identifying patients at risk for adverse psychological outcome two months after critical illness. Prior to wider clinical use, external validation is needed.

## Introduction

Intensive care unit (ICU) survivors are at risk of developing a number of physical, psychological and cognitive problems following critical illness. Different follow-up strategies have been studied and employed to reduce post-ICU morbidity [1-3]. As some patients recover uneventfully from critical illness, offering all ICU survivors resource-intensive interventions may be a costly way of reducing post-ICU problems [2]. A number of studies have reported risk factors for physical and psychological dysfunction after intensive care [4-7]. However, there is currently no agreement on how to determine the individual patient's risk for problems after critical illness. Swedish guidelines suggest follow-up for patients with an ICU length of stay longer than four days [8], a selection that has not been thoroughly evaluated. While the United Kingdom National Institute of Clinical Excellence (NICE) guidelines [9] suggest considering risk factors for impaired physical and psychological outcome post ICU, there is little guidance on how the bedside-clinician should use these recommendations. This study focused on psychological problems after critical illness. In order to optimize treatment of ICU survivors and to improve resource allocation in ICU follow-up, a method for identifying patients in need of help in psychological recovery after intensive care would be of value. The aim of this study was to develop a screening instrument for use at ICU discharge, identifying patients at risk for later post-traumatic stress, anxiety or depression.

## Materials and methods

This prospective cohort study was conducted in the Karolinska University Hospital Solna in Sweden, a tertiary care hospital. The General ICU is a mixed adult medical and surgical 13-bed ICU with a nurse:patient ratio of 1:1. Around 900 patients with surgical or medical diagnoses are admitted to the ICU yearly. The study was approved by the Regional Ethical Review Board in Stockholm (EPN Stockholm dnr 2010/206-31/1).

### Participants

**Figure 1:**
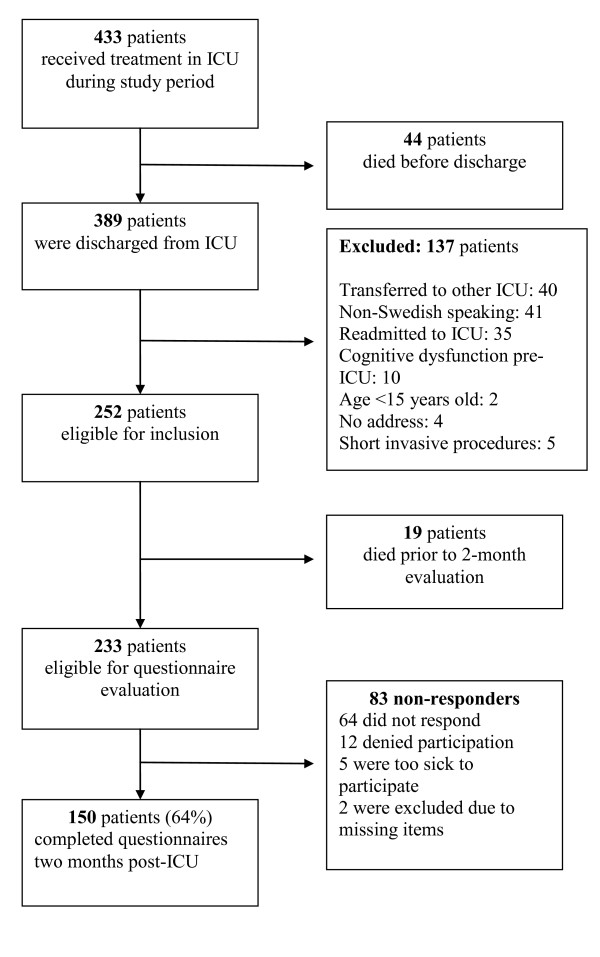
**Flow chart of patient inclusion**.

All patients discharged from the General ICU during a six-month period in 2011 were consecutively enrolled in the study (Figure 1). Patients transferred to intensive care units in other hospitals, non-Swedish speaking patients, those with previous cognitive impairment and homeless patients with no formal address were excluded. Also, patients admitted shortly for invasive procedures, such as placement of epidural catheters or central venous lines were excluded. For patients readmitted to the ICU during the study period, only data from the final admission were used in the analysis. Written informed consent was obtained from all participants in the study.

### Potential risk factors

A literature review was performed to identify previously described risk factors for psychological morbidity after critical illness [4-7] and specific factors influencing psychological recovery in general [10]. Also, the NICE guideline 83 for rehabilitation after critical illness was studied [9]. Previously described risk factors from the literature review are shown in Additional file 1. Potential risk factors were selected after this literature review and a consensus discussion with ICU clinicians running a follow-up clinic, together with a clinical psychologist specialized in traumatic stress. The selection and definition of potential risk factors was based on: a) the applicability for a heterogeneous critically ill population, b) the feasibility for ICU clinicians to assess the risk factor and c) a fair possibility of assessment before the patient left the ICU. The review and consensus discussion rendered 21 potential risk factors described in Additional file 2.

Patient characteristics included age, gender, marital status, parenthood (with children <18 years of age), level of education, occupational status and presence of pre-existing diseases or previous psychological problems. Variables related to the ICU stay were Simplified Acute Physiology Score (SAPS) III, main ICU diagnosis (divided into trauma, surgical diagnoses, medical diseases or infections), ICU length of stay, type and duration of sedative and opiate infusions, duration of ventilator support, presence of delirium, hallucinations, agitation, patients' mood (depressive signs or symptoms), ability to take their own initiative and lack of social support.

### Data collection

Data regarding patient characteristics were collected from the patient him/herself or the next of kin, together with information obtained from medical charts. To score pre-existing diseases, the Charlson Co-morbidity Index (CCI) [11] was used. The index was originally developed to predict the 10-year mortality for patients with somatic diseases, such as heart disease or cancer. Each condition is assigned a score of 1, 2, 3 or 6, depending on the mortality risk associated with this condition. Information regarding previous psychological problems was collected from the medical charts and was defined as one of the following: a) a history of prior episodes of depression or anxiety, b) a psychiatric diagnosis in the medical charts, and c) documented alcohol or drug abuse.

Data regarding patients' ICU stay were obtained from the local patient data management system and medical charts and information gathered by the patient's nurse. Three times daily (once per work shift), the patient's nurse evaluated the presence of delirium, hallucinations, agitation, patients' mood (depressive signs) and ability to take the initiative. The presence of delirium was assessed with the Confusion Assessment Method for Intensive Care Unit [12]. Hallucinations were assessed by asking the patients if they experienced visual or auditory sensations without a sense of reality. Agitation was assessed with Motor Activity Assessment Scale (MAAS) [13]. Agitation was considered present if MAAS was >4, if the patient was aggressive and uncooperative, with signs of panic or confusion. The presence of agitation was based on observations made by the patient's nurse, who if necessary reviewed the medical chart. For communicative patients, the nurse asked the patient if he/she felt depressed. For patients verbally unable to express their feelings (for example, tracheostomy) or general fatigue, other signs of depression were noted (by answering the question "Does the patient appear apathetic, low or express hopelessness?"). Ability to take initiative was a factor considered to reflect patients' ability to influence outcome. A patient was considered able to take initiative if he or she verbally or non-verbally took initiative in any activity. Patients with no visits from next of kin during the ICU stay were considered not having social support.

### Outcome measures

Two months after individual ICU discharge, patients received the Post-Traumatic Stress Symptom scale (PTSS)-10 questionnaire [14] and Hospital Anxiety and Depression Scale (HADS) [15] by postal mail. Two weeks after this, non-responders received a reminder by phone. If there was still no response, a reminder letter was sent to the participants after four weeks. Psychological evaluation at two months was considered an appropriate time point, since early treatment may prevent or reduce the severity of post-traumatic stress [10].

PTSS-10 is a validated, self-administered screening instrument used for measuring symptoms of post-traumatic stress [14], mostly in critically ill patients [16,17]. Ten common symptoms of post-traumatic stress are rated on a scale, depending on how frequently they appear. Each symptom is graded from 1 (never) to 7 (always) with the maximum score of 70. A total score above 35 is suggestive of post-traumatic stress disorder [14,16].

Anxiety and depression were measured with the HADS, using two separate subscales (maximum subscale score 21) [15]. HADS has been validated in a Swedish sample [18]. Subscale scores of 8 or above indicate possible cases and ≥11 indicates caseness of anxiety or depression [15]. We defined adverse psychological outcome after ICU as PTSS-10 score >35 and/or HADS subscale score ≥8.

### Statistical analysis

Patient characteristics were presented with proportions (%) for categorical variables and with medians and interquartile ranges for continuous variables. Fisher's Exact Test was used to compare categorical data and the Mann Whitney U-test to compare continuous data between responders and non-responders. *P*-values below 0.05 were considered to indicate statistical significance. To predict the probability of adverse psychological outcome, the 21 variables consisting of demographic data and information gathered at ICU discharge were examined for univariate associations. The univariate relationship between the risk factors and psychological adverse outcome was assessed in a logistic regression model with one covariate at a time. Variables with a *P*-value less than 0.10 were included in a multivariable logistic regression model. The area under the receiver operating characteristics (AUROC) curve was utilized as a measure of overall accuracy of the predictive model. The predictors were removed one at a time, and the AUROC curve was recalculated each time [19]. To further evaluate the predictive accuracy of the screening instrument, the AUROC curve was internally cross-validated in 1,000 bootstrap samples [20]. As Swedish guidelines recommend ICU length of stay more than four days as a cut-off for follow-up of patients [8], we also performed an analysis of ICU length of stay as a predictor of adverse psychological outcome, with AUROC curve analysis. The analyses were performed using Stata version 12 (StataCorp, College Station, TX, USA) and IBM SPSS version 20.0 (IBM, Chicago, IL, USA).

## Results

Among 433 patients admitted to the General ICU during the study period, 252 patients met the inclusion criteria, and 150 (64%) completed the questionnaires (Figure 1). There were no significant differences in patient characteristics between responders and non-responders besides that patients responding to the questionnaires were older (median age 59 versus 46, *P *< 0.05) and had more pre-existing diseases (median CCI 1.0 versus 0, *P *< 0.05). In responders, 46 patients (31%) reported adverse psychological outcome, defined as PTSS-10 >35 and/or HADS subscales ≥8, two months after ICU discharge (Table 1).

**Table 1:** Patient and treatment characteristics for responders with high scores and low scores in questionnaires

	Adverse psychological outcome (*n *= 46)	No adverse psychological outcome (*n *= 104)
Age	54 (38 to 65)	60 (45 to 68)
Women	46%	37%
Pre-existing diseases (CCI)	1 (0 to 3)	1 (0 to 2)
Previous psychological problems	37%	13%
Diagnosis group:		
Trauma	22%	27%
Surgical complications	26%	32%
Medical diseases	26%	14%
Infections	26%	27%
SAPS III	55 (41 to 63)	55 (42 to 64)
ICU length of stay (days)	1.6 (1 to 5)	1.5 (1 to 4)
Mechanical ventilation (days)	0 (0 to 2)	0 (0 to 2)

High scores were regarded as adverse psychological outcome and were defined as PTSS-10 > 35 and/or HADS subscale score ≥ 8. Low scores, no adverse psychological outcome, were defined as PTSS-10 ≤ 35 and/or HADS subscale score ≤ 7.

Data were presented as median (interquartile range 25 to 75) and proportions % where appropriate. CCI, Charlson Comorbidity Index; ICU, Intensive care unit; SAPS III, Simplified Acute Physiology Score III

### The predictive model

**Figure 2:**
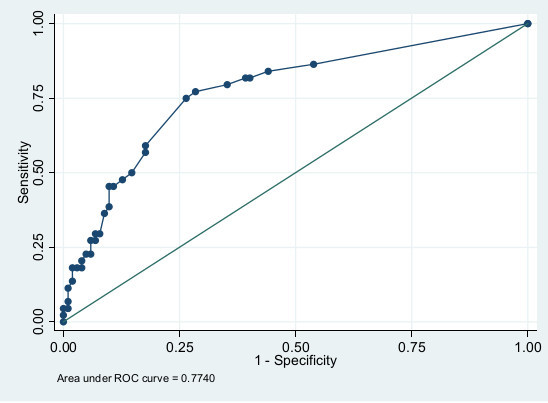
**Receiver operating characteristics (ROC) curve for the predictive model**.

**Figure 3:**
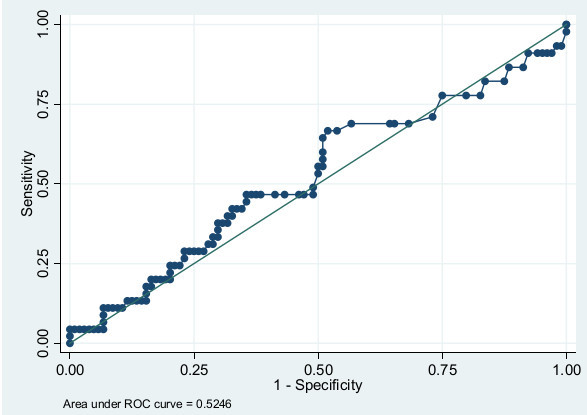
**ROC curve for ICU length of stay as a predictor of adverse psychological outcome**.

All 21 variables had a prevalence rate above 10%. The univariate associations of the risk factors are shown in Additional file 2. Seven variables with a *P*-value below 0.10 were included in a multivariable logistic regression model. "Diagnosis group" was removed from the model as this reduced the area under the curve by less than one percentage point and therefore it was not considered essential from the clinical viewpoint. The six variables predictive of adverse psychological outcome after critical illness were: major pre-existing diseases (defined as CCI >3), having children younger than 18 years of age, previous psychological problems, in-ICU agitation, being unemployed or on sick-leave at ICU admission and appearing depressed in ICU. The variables are presented with regression coefficients, odds ratio and 95% confidence intervals (CI) in Table 2. Pre-existing diseases, as measured with total CCI, were dichotomized with a cut-off of CCI >3, as this cut-off showed a distinct divergence in the predictive value. The regression coefficient of each variable was equivalent to its associated probability for adverse psychological outcome. In order to make the coefficients more manageable, they were multiplied by 25 and named "risk scores". The total risk score was almost linear in relation to and equal to the risk of adverse psychological outcome in percent (Table 2). The accuracy of the final model, assessed as the AUROC curve, was 0.77 (95% CI: 0.69 to 0.86) (Figure 2). When testing ICU length of stay as a predictor of adverse psychological outcome, the AUROC curve was only 0.53 (95% CI: 0.42 to 0.62) (Figure 3). In a *post-hoc *analysis, we categorized patients according to their probability of having adverse psychological outcome in low risk (0 to 29%), moderate risk (30 to 59%), and high risk (60 to 100%) groups. The proportion of patients in each of the risk groups and the actual prevalence of adverse psychological outcome are reported in Table 3.

**Table 2:** Regression coefficients, odds ratios and risk scores for predictors of adverse psychological outcome

Predictors	Regression coefficient	Odds ratio	95% CI	**Risk score = **Regression coefficient × 25
Pre-existing diseases (CCI >3)	2.02	7.52	2.01 to 28.1	**50.5**
Parent to children <18 years	1.26	3.51	1.39 to 8.89	**31.5**
Previous psychological problems	1.14	3.13	1.12 to 8.76	**28.5**
In-ICU agitation	0.8	2.23	-0.80 to 6.22	**20**
Unemployed/sick-leave at ICU admission	0.48	1.62	-0.54 to 4.88	**12**
Appeared depressed in ICU	0.30	1.35	-0.55 to 3.31	**7.5**

In order to make risk scores in the predictive screening instrument easier to compute the coefficients have been multiplied by 25 and named "risk score". CCI, Charlson Co-morbidity Index; CI, Confidence interval; ICU, Intensive care unit

**Table 3:** Risk group probabilities and actual prevalence of adverse psychological outcome in each group

Risk group	(risk in %)	Percent of patients in cohort	Percent of patients with observed adverse psychological outcome
Low risk	0 to 29	57%	12%
Moderate risk	30 to 59	30%	50%
High risk	60 to 100	13%	63%

### Cross-validation

The AUROC curve in this sample of patients was estimated to be 0.72 (95% CI: 0.56 to 0.83), after cross-validation in 1,000 bootstrap samples.

## Discussion

To our knowledge, this is the first description, in a mixed ICU population, of an early screening method - for use already at ICU discharge - to assess the individual patient's risk for later adverse psychological outcome, defined as moderate to high levels of post-traumatic stress, anxiety or depressive symptoms.

### Selection of predictors

Six predictors were included in the final screening instrument, each with a relative probability for later psychological problems. The association between higher CCI and psychological problems after ICU stay in our study are congruent with data from previous studies, in which chronic medical illness has been found to be associated with depression [21,22], anxiety disorders [23,24] and low health-related quality of life [25,26]. Either patients with chronic medical illness already had more psychological problems prior to the ICU stay or the combination of pre-existing disease and being critically ill might have worsened their psychological well-being. Regardless of the association with chronic medical illness, treatment of identified significant psychological problems may improve patients' well-being and quality of life [24]. Adverse psychological outcome after critical illness and ICU stay was more common in patients with children under the age of 18. Parenthood has previously been found to be a predictor of post-traumatic stress disorder [27]. We speculate that the increased psychological morbidity found in parents of young children might be due to a stronger burden of responsibility (as younger children are normally more dependent) combined with the vulnerability experienced after a life-threatening injury or illness. Our finding that previous psychological problems and unemployment increase the risk of psychological morbidity in ICU patients is corroborated by other studies [4,23,28]. In contrast to some studies [29-31], female gender was not an important predictor for psychological morbidity, after including previous psychological problems as a predictor, which is more common in women [32,33].

Delirium as a whole was not found to be a predictor of adverse psychological outcome. At the time of the study, delirium screening performed by the ICU nurses did not discriminate between hypoactive and hyperactive delirium, precluding analysis of delirium subtypes. Agitated behavior in the ICU, however, appeared to be a significant predictor for adverse psychological outcome as found in another study [34]. Possibly, delirium screening with separation of subtypes would have identified agitated delirium as a predictor for adverse psychological outcome. We used the MAAS for assessing agitation. For units using the Richmond Agitation and Sedation Scale (RASS) [35], we suggest that MAAS levels of 5 ("Agitated)" or more may translate fairly well to a RASS level of +2 ("Agitated") or more. With this stated, we are not aware of any study validating this translation.

In previous studies, depressive symptoms post-ICU, assessed at hospital discharge [36] or a few months after ICU discharge [7,37] have proven to be strong predictors for later development of depression. In our study, reporting or exhibiting depressive signs or symptoms already in the ICU was found to be predictive of adverse psychological outcome two months later. A more formal psychiatric assessment of depressive symptoms might have improved the validity of this finding but would be more resource-intensive, require longer time than our suggested assessment and could be demanding for a number of patients at the time for ICU discharge. Depressive symptoms as a predictor of subsequent psychological morbidity have been described previously [38].

Interestingly, four of the predictors included in the screening instrument were not specific for ICU patients. Some stressors may be unique for the ICU situation but being seriously ill may be traumatic irrespective of ICU admission. Critical illness may add an extra burden on already psychologically or physically vulnerable individuals and serve as a trigger for negative emotional reactions [39]. Another explanation may be that patients with multiple predictors already had on-going psychological symptoms prior to the ICU stay. Almost all patients were admitted to the ICU due to acute illness or injury, precluding baseline assessment.

It is likely that other factors than those described in our study play a role in the trajectory of psychological well-being and morbidity. We did not assess genetic or biological factors, which may influence the psychological outcome after a traumatic event. Factors promoting resilience, such as self-esteem, trust and resourcefulness, may be important in preventing persistent stress reactions but might be more difficult to assess by untrained ICU clinicians, for whom we aimed to develop the screening instrument. Primarily, vulnerability factors were investigated in this study. Some clinically relevant risk factors for psychological morbidity, such as traumatic memories [10,36,40] or personality traits [41], were not included since they were considered being difficult for ICU staff to assess at ICU discharge. With this stated, prediction of psychological problems with the six risk factors was fairly good and the instrument may be a starting point for decision-making regarding which patients to consider for early post-ICU follow-up. A number of patients might have an intermediate risk for psychological problems and for this group of patients, post-ICU instruments assessing ICU experiences [42] or personality trait [43] could be valuable in further assessment.

### Clinical application and feasibility of the predictive screening instrument

**Figure 4:**
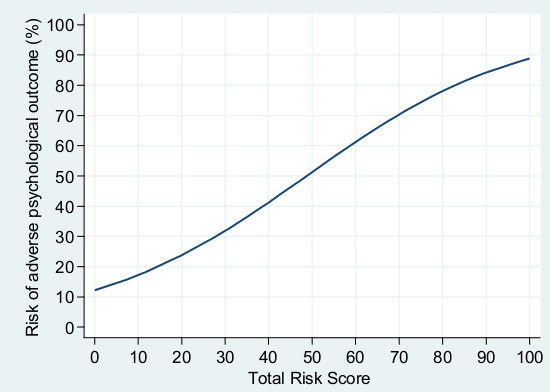
**Curve of the total risk score and the corresponding risk for adverse psychological outcome**.

Our main aim was to develop an instrument manageable for ICU clinicians to identify patients at risk of adverse psychological outcome. The predictive screening instrument consists of Table 4 and 5 and Figure 4. When using the screening instrument, the first step is to assess which predictors the patient has at the time of discharge to the ward (Table 4). Each predictor corresponds to a given risk score (the coefficient in the logistic regression multiplied by 25). The total risk score, the sum of individual risk scores, is then calculated. For example, if the patient has major pre-existing diseases assessed as the Charlson Comorbidity Index >3 (Table 5) and is on sick-leave at ICU admission, the total risk score for the patient is 50.5 +12 = 62.5. This risk score corresponds with a risk probability and can be plotted on the risk score-probability curve in the screening instrument (Figure 4), which in this case indicates a probability of approximately 60% for adverse psychological outcome in the individual patient. As stated in the results, the obtained total risk score is almost equal to the risk probability in percent, in our view making interpretation of the obtained risk score relatively straightforward even without plotting values on the curve.

**Table 4:** The screening instrument.

	If yes, add the scores
**1. The patient has major pre-existing diseases (Charlson Co-morbidity Index >3)**	50.5
**2. The patient has children <18 years of age**	31.5
**3. The patient has previous psychological problems** **Defined as prior episodes of depression, anxiety or having other psychiatric diagnoses and/or documented alcohol or drug abuse. If possible, ask the patient or his/her next-of-kin**.	28.5
**4. The patient was unemployed or on sick-leave at ICU admission**	12
**5. The patient was agitated in ICU** **MAAS >4, defined as aggressive behavior with confusion or panic**.	20
**6. The patient appeared depressed in ICU** **Defined as sadness, apathy or feelings of hopelessness. If possible, ask the patient if he/she feels depressed**.	7.5

**Total risk score:**	

After each predictor has been evaluated, the total risk score is calculated. ICU, Intensive Care Unit; MAAS, Motor Activity Assessment Scale [13].

**Table 5:** Identify any pre-existing disease and summarize the total Charlson Co-morbidity Index score (CCI).

Medical conditions	Scores
Myocardial infarct	1
Congestive heart failure	1
Peripheral vascular disease	1
Cerebrovascular disease	1
Dementia	1
Chronic pulmonary disease	1
Connective tissue disease	1
Ulcer disease	1
Mild liver disease	1
Diabetes	1
Hemiplegia/paraplegia	2
Moderate or severe renal disease^a^	2
Diabetes with end organ damage^b^	2
Any tumor	2
Leukemia/lymphoma	2
Moderate or severe liver disease	3
Metastatic solid tumor	6
AIDS	6

**Summarized CCI-score**	

A total score above three (CCI>3), is regarded as presence of major pre-existing diseases in Table 4.

^a^Patients on dialysis, with uremia or who have had kidney transplantation. ^b^Patients with retinopathy, neuropathy, nephropathy, with juvenile onset or previous episodes of ketoacidosis or hyperosmolar coma.

We performed a *post-hoc *categorization of patients in three risk groups. This division of patients into low risk, moderate risk and high risk groups might possibly aid ICU clinicians in deciding potential treatment policies. According to our model and suggested risk groups, 57% of patients would be considered having low risk (0 to 29%) for psychological problems and, therefore, might be excluded from early, active, in-hospital follow-up. By excluding patients with low risk for future problems, a smaller number of patients remain for more resource-intensive assessment and treatments by trained clinicians. Such assessment may include an evaluation of what problems are new-onset and related to the recent episode of critical illness and ICU stay. When comparing the predictive accuracy of the screening instrument with the current Swedish guidelines recommending follow-up for patients with an ICU length of stay of more than four days, prediction was better with the screening instrument. The AUROC curve for the predictive instrument was 0.77 (0.72 after cross-validation), which is fair in discriminating psychologically sound patients from those with poor psychological recovery. When using ICU length of stay as a predictor of adverse psychological outcome, the AUROC curve was 0.53, almost equal to chance.

We only evaluated the possibility to predict adverse psychological outcome. Physical, psychological and cognitive problems are sometimes [44], but not always, linked and may require different treatments. Structured models or screening instruments for predicting physical and cognitive problems would be valuable to use in parallel with a psychological screening instrument.

### Limitations

The main limitation of our study is that it is a single center study and external validity has not yet been verified. There may be a divergence of the risk factors' predictive effect in different populations which may affect the predictive value of the model in other settings [45]. Even though 150 patients participated in the study, a larger sample size would likely have improved the accuracy of the predictive model. Our cohort was a mixed ICU population with a wide range of diagnoses, which, on the other hand, could be considered a strength.

Cross-validation confirmed the robustness of the model within the study cohort and the preliminary model appeared to perform significantly better than the Swedish recommendation of four days in the ICU as a cutoff. Nevertheless, our study represents only the first step in the development of a risk model. The model needs to be validated in other ICU populations before more widespread clinical use.

Our hospital facilitates both early ICU follow-up, including brief recapitulation of the ICU stay and giving patients an ICU diary, procedures that may potentially affect the prevalence of later adverse psychological outcome. Hence, the outcome and the predictive performance of the model could be different in hospitals without any follow-up service. The assessment of depressive symptoms before ICU discharge could possibly have been performed with validated instruments, or by trained psychologists. In our view, however, this might have made assessment of this single risk factor too cumbersome for the critically ill patient and also would have limited the practicability of a future screening instrument and, therefore, this risk factor was assessed by the patient's ICU nurse.

The proportion of patients with adverse psychological outcome depends on the selection of screening instruments and chosen cut-off levels. As the cut-off level for HADS we used subscale scores ≥8, the cut-off suggesting possible anxiety or depression rather than 11, the cut-off for likely presence of clinical anxiety or depression. Our choice may increase the number of cases for follow-up identified by the screening instrument compared with the more conservative cut-off of 11. The first step in follow-up after initial screening is a confirmation by trained clinicians, rather than pharmacological treatment or other therapies. Initial false-positive patients will thus not be directly exposed to any significant risk.

Finally, while ICU length of stay appears not to predict psychological problems effectively, it may possibly have a better predictive value for other post-ICU problems, such as physical impairment.

## Conclusion

The screening instrument for predicting psychological morbidity after critical illness and general intensive care developed in this study may assist ICU clinicians in identifying patients at risk for adverse psychological outcome after critical illness. The model needs external validation in other ICU populations before it can be applied more generally.

## Key messages

• A preliminary instrument manageable for ICU clinicians to identify patients at risk for later psychological problems, already in the ICU, may facilitate and improve resource allocation in ICU follow-up.

• Major pre-existing diseases, having children younger than 18 years of age, previous psychological problems, in-ICU agitation, being unemployed or on sick-leave at ICU admission and appearing depressed in the ICU appear to be important predictors for psychological morbidity after critical illness in a mixed ICU population.

## Abbreviations

AUROC: Area under receiver operating characteristics; CCI: Charlson Co-morbidity Index; CI: Confidence interval; ICU: Intensive care unit; HADS: Hospital Anxiety and Depression Scale; MAAS: Motor Activity Assessment Scale; NICE: National Institute of Clinical Excellence; PTSS-10: Post-Traumatic Stress Symptom scale -10; SAPS: Simplified Acute Physiology Score.

## Competing interests

The authors declare that they have no competing interests.

## Authors' contributions

AS, ÖS and PS designed the study. AS and PS reviewed the literature. AS and EH were responsible for the data collection. AS and MB performed the statistical analyses. AS and PS wrote the draft and all other authors critically revised the manuscript and approved the final version for publication.

## Supplementary Material

Additional file 1 **Identified risk factors in the literature review and potential risk factors included in the prediction study**. Click here for file

Additional file 2 **Description of risk factors and their univariate associations with adverse psychological outcome**. Click here for file
