# Supplementary material for: Developing an early screening instrument for predicting psychological morbidity after critical illness
Source: Crit Care. 2013 Sep 24;17(5):R210. doi: 10.1186/cc13018 (PMC4057163; doi:10.1186/cc13018)
Supplement: Additional file 1 — Identified risk factors in the literature review and potential risk factors included in the prediction study. [file cc13018-S1.DOC]

**Additional file 1.** Supplement for web-publication

Identified risk factors in the literature review and potential risk factors included in the prediction study

| **Study** | **Identified risk factors** | **Potential predictors in the study** |
| --- | --- | --- |
| Davydow et al. (1) | Pre-ICU psychopathology  Pre-ICU physical function  Early symptoms of depression  Longer ICU stay  Duration of sedation  Duration of mechanical ventilation  Increased in-ICU Benzodiazepine use  Post-ICU traumatic memories | Included  Indirectly assessed as comorbidity  Included  Included  Included  Included  Included  Not applicable at ICU discharge |
| Davydow et al. (2) | Pre-ICU depression  Pre-ICU alcohol abuse  Major comorbidity  Level of education  Longer ICU stay  Duration of mechanical ventilation  PAC insertion  Decreased quality of life 3 months after ICU  Receiving blood products in the first 24h of hospitalization | Included  Included in “Psychological problems”  Included  Included  Included  Included  Proxy: Severity of illness (SAPS III)  Not applicable at ICU discharge  Proxy: SAPS III |
| Hopkins et al. (3) | Younger age  Female sex  Pre-ICU alcohol abuse  Oxygen tension /oxygen fraction  Mechanical ventilation  Post-ICU depression/anxiety | Included  Included  Included in “Psychological problems”  Considered not practicable to assess  Included  Not applicable at ICU discharge |
| Davydow et al. (4) | Female sex  Pre-ICU functioning  Poor recall from ICU  Memories of stressful events in ICU  Early depressive symptoms  Post ICU PTSD symptoms  Cognitive impairment six months post ICU | Included  Sick leave pre-ICU, major comorbidity  Not applicable at ICU discharge  Not possible to assess at ICU discharge  Included  Not possible to assess at ICU discharge  Not possible to assess at ICU discharge |
| Van der Kolk et al. (5) | Pre-ICU psychopathology  Trauma  Poor social network  Biology  Neuroticism | Included  Included  Included  Considered not feasible to assess  Difficult to assess at ICU discharge |
| NICE guideline CG 83  suggestions (6) | Recurrent nightmares  Intrusive memories of trauma  Anxiety or panic attacks  Avoiding to talk about the situation | Difficult to quantify at ICU discharge  Difficult to assess at ICU discharge  Agitation used as proxy  Difficult to assess at ICU discharge |

ICU=Intensive Care Unit; PTSD=Post-traumatic stress disorder; SAPS III= Simplified Acute Physiology Score III

1. Davydow DS, Katon WJ, Zatzick DF. Psychiatric morbidity and functional impairments in survivors of burns, traumatic injuries, and ICU stays for other critical illnesses: a review of the literature. *Int Rev Psychiatry* 2009;21(6):531-538

2. Davydow DS, Zatzick DF, Rivara FP, et al. Predictors of posttraumatic stress disorder and return to usual major activity in traumatically injured intensive care unit survivors. *Gen Hosp Psychiatry* 2009;31(5):428-435

3. Hopkins RO, Key CW, Suchyta MR, et al. Risk factors for depression and anxiety in survivors of acute respiratory distress syndrome. *Gen Hosp Psychiatry* 2010;32(2):147-155

4. Davydow DS, Gifford JM, Desai SV, et al. Depression in general intensive care unit survivors: a systematic review. *Intensive Care Med* 2009;35(5):796-809

5. Van Der Kolk B, McFarlane A, Weisaeth L: Traumatic stress: The effects of overwhelming experience on mind, body and society. New York, Guilford Publications, 2006

6. National Institute for Health and Clinical Excellence: Rehabilitation after critical illness. Available at: http://www.nice.org.uk/CG83. Accessed Feb 10th, 2013
