# Supplementary material for: Developing an early screening instrument for predicting psychological morbidity after critical illness
Source: Crit Care. 2013 Sep 24;17(5):R210. doi: 10.1186/cc13018 (PMC4057163; doi:10.1186/cc13018)
Supplement: Additional file 2 — Description of risk factors and their univariate associations with adverse psychological outcome. [file cc13018-S2.DOC]

**Additional file 2.** Supplement for web-publication

Description of predictors and their univariate associations with adverse psychological outcome

| **Predictors** | **Description** | **Univariate associations (p-values)** |
| --- | --- | --- |
| Age |  | 0.247 |
| Gender | 0=Men  1=Women | 0.248 |
| Marital status | 0=No life partner  1=Having lift partner | 0.655 |
| Parenthood | 0=No children/ children older than 18 years 1=Having children younger than 18 years | 0.024* |
| Education level | 1=Elementary school  2=Senior high school  3=College/university | 0.402 |
| Occupational status pre-ICU | 0=On sick leave or unemployed  1=Working, student, retired or maternity leave | 0.025* |
| ICU length of stay |  | 0.380 |
| SAPS III |  | 0.902 |
| Diagnosis | 0=Infection, surgery or medical diseases  1=Trauma | 0.083* |
| Comorbidity | 0=CCI ≤ 3  1=CCI > 3 | 0.002* |
| Psychological problems pre-ICU | 0=No previous psychological problems  1= Previous psychological problems | 0.001* |
| Propofol use | 0=Administered for less than 24 hours  1=Administered for 24 hours or more | 0.495 |
| Midazolam use | 0=Administered for less than 24 hours  1=Administered for 24 hours or more | 0.678 |
| Morphine use | Days of Morphine use | 0.809 |
| Ventilator treatment | 0=Ventilator treatment for less than 24 hours  1= Ventilator treatment for 24 hours or more | 0.906 |
| Delirium | 0=No in-ICU delirium  1=Presence of delirium in ICU | 0.845 |
| Hallucinations | 0=No hallucinations in ICU  1=Presence of hallucinations in ICU | 0.332 |
| Agitation | 0=No in-ICU agitation  1=Presence of in-ICU agitation | 0.058* |
| Ability to initiative | 0=Does not take initiatives in ICU  1=Takes own initiative in ICU | 0.857 |
| Appears depressed | 0=Does not appear depressed in ICU  1=Appears depressed in ICU | 0.091* |
| Lack of social support | 0=No family present in ICU  1=Family present in ICU | 0.409 |

*Included in the multivariate logistic regression model . CCI=Charlson Comorbidity Index; ICU=Intensive care unit; SAPS III= Simplified Acute Physiology Score III.
